# Psychometric Evaluation of the Overexcitability Questionnaire-Two Applying Bayesian Structural Equation Modeling (BSEM) and Multiple-Group BSEM-Based Alignment with Approximate Measurement Invariance

**DOI:** 10.3389/fpsyg.2015.01963

**Published:** 2015-12-24

**Authors:** Niki De Bondt, Peter Van Petegem

**Affiliations:** Department of Instructional and Educational Sciences, Faculty of Social Sciences, University of AntwerpAntwerp, Belgium

**Keywords:** Bayesian structural equation modeling, Overexcitability Questionnaire-Two (OEQ-II), approximate measurement invariance, alignment optimization method, Dabrowski's Theory of Positive Disintegration, psychometrics

## Abstract

The Overexcitability Questionnaire-Two (OEQ-II) measures the degree and nature of overexcitability, which assists in determining the developmental potential of an individual according to Dabrowski's Theory of Positive Disintegration. Previous validation studies using frequentist confirmatory factor analysis, which postulates exact parameter constraints, led to model rejection and a long series of model modifications. Bayesian structural equation modeling (BSEM) allows the application of zero-mean, small-variance priors for cross-loadings, residual covariances, and differences in measurement parameters across groups, better reflecting substantive theory and leading to better model fit and less overestimation of factor correlations. Our BSEM analysis with a sample of 516 students in higher education yields positive results regarding the factorial validity of the OEQ-II. Likewise, applying BSEM-based alignment with approximate measurement invariance, the absence of non-invariant factor loadings and intercepts across gender is supportive of the psychometric quality of the OEQ-II. Compared to males, females scored significantly higher on emotional and sensual overexcitability, and significantly lower on psychomotor overexcitability.

## Introduction

### Overexcitability within dabrowski's theory of positive disintegration

Dabrowski (1902–1980), a Polish psychiatrist and psychologist, developed the Theory of Positive Disintegration, which centers on heightened excitability in individuals, as well as on their drive, and their urge to resist conformity and complacency (Daniels and Piechowski, 2009). According to Dabrowski (1964; 1972; Mendaglio, 2008), personality is achieved through a process of positive disintegration, which begins with the disintegration of a primitive mental organization aimed at meeting biological needs and conforming to societal norms. Reintegration subsequently takes place at a higher level of human functioning, as characterized by autonomy, authenticity, and empathy. Achieving the highest level of human development—or enacting the personality ideal—depends on the developmental potential of an individual, which is determined by the individual's level of innate heightened excitability (overexcitability) and the presence of specific talents, abilities, and autonomous inner forces that cultivate growth (dynamisms).

According to Dabrowski, the developmental potential of an individual depends in part on the extent and nature of psychic intensity. Dabrowski uses the term “overexcitability” to refer to an above average responsiveness to stimuli, due to heightened sensitivity of the central nervous system, which generates a different, more intense, and more multi-faceted experience of internal and external reality (Dabrowski, 1964, 1972; Mendaglio, 2008). Dabrowski distinguishes five forms of overexcitability. Psychomotor overexcitability represents “a surplus of energy or the expression of emotional tension through general hyperactivity” (Silverman, 2008, p. 160). Manifestations include an abundance of physical energy, work addiction, nervous habits, rapid speech, love of movement, impulsiveness, competitiveness, and an urge to action. Sensual overexcitability involves enhanced receptivity of the senses, aesthetic appreciation, sensuality, and pleasure in being the center of attention. Imaginational overexcitability is characterized by a capacity to visualize events very well, as well as by ingenuity, fantasy, a need for novelty and variety, and poetic and dramatic perception. Intellectual overexcitability is characterized by an intensified activity of the mind, as well as by asking penetrating questions, reflective thought, problem solving, searching for truth and understanding, conceptual and intuitive integration, and an interest in abstraction and theory. Emotional overexcitability involves an intense connectedness with others, as well as the ability to experience things deeply, strong affective and somatic expressions, sensitivity in relationships, responsiveness to others, and well-differentiated feelings toward self (Silverman, 2008; Daniels and Piechowski, 2009). Dabrowski considers the last three forms of overexcitability essential to advanced personality development (Dabrowski, 1972; Mendaglio, 2008).

### The overexcitability questionnaire-two and its psychometric properties

Falk et al. (1999) developed a self-report questionnaire to measure the degree and nature of overexcitability. The Overexcitability Questionnaire-Two (OEQ-II) continues to be used primarily in research positioned within the domain of giftedness. Numerous studies on intensity in gifted and non-gifted students have demonstrated associations of giftedness with intellectual (Bouchet and Falk, 2001; Tieso, 2007a; Siu, 2010; Carman, 2011; Harrison and Van Haneghan, 2011; Wirthwein and Rost, 2011; Wirthwein et al., 2011; Van den Broeck et al., 2014), imaginational (Tieso, 2007a; Siu, 2010; Carman, 2011; Harrison and Van Haneghan, 2011), and emotional (Bouchet and Falk, 2001; Siu, 2010) overexcitability. The OEQ-II has been translated into Turkish, Chinese, Korean, Spanish, French, and Dutch (Falk et al., 2008; Siu, 2010; He and Wong, 2014; Van den Broeck et al., 2014; Botella et al., 2015). Empirical research has revealed that emotional, intellectual, and imaginational overexcitability are important indicators and predictors of advanced personality development (Falk and Miller, 2009).

Despite the rising tendency in empirical research to use the OEQ-II as a supplementary measure of dispositional traits, the instrument has been validated in a limited way. Falk et al. (1999, p. 2) developed the easily administered and scored OEQ-II by incorporating the results of numerous prior studies on hyperexcitability, “including responses to deep reflex stimulation, open-ended responses to verbal stimuli, assessment in autobiographical material, and an open-ended questionnaire.” The authors investigated the structural validity of the questionnaire via principal components factor analysis using varimax rotation. A stable and conceptually clear five-factor structure was retrieved with factor loadings above 0.50, and good internal consistency among the items indicative of the same factor was found for the two samples under study (Falk et al., 1999).

Van den Broeck et al. (2014) investigated the factorial structure of the OEQ-II (Dutch translation), using exploratory structural equation modeling (ESEM; Asparouhov and Muthén, 2009) with weighted least squares estimation and oblique target rotation. The highly restrictive independent clusters model used in the confirmatory factor analysis (CFA), in which each indicator is allowed to load on only one factor and all non-target loadings are constrained to zero (Marsh et al., 2009), led to model rejection. Model testing using ESEM, in which the five correlated overexcitability factors were measured by each of the 50 items, yielded a partly satisfactory model fit. Modification indices were inspected in order to improve the model by including two residual covariances, ultimately leading to an acceptable fit to the data. This study further examined measurement invariance across intelligence levels and gender using an ESEM-Within-CFA approach (Marsh et al., 2013). This analysis established partial strict measurement invariance of the OEQ-II scores across the different groups. The researchers concluded that the non-invariant parameters do not considerably affect group comparisons because of their small proportionality.

Warne (2011) also investigated measurement invariance of the OEQ-II scores across gender using a multi-group CFA approach and maximum likelihood estimation, but the study could not establish metric invariance.

Botella et al. (2015) examined the structural validity of the French OEQ-II using CFA and maximum likelihood estimation. Instead of freeing “an important number of cross-loadings and residual covariances” (Botella et al., 2015, p. 211), the researchers first reduced the instrument to a 35-item version and concluded that a model with “five correlated factors with residual covariances” yields a better fit to the data compared to a “one second-order factor” model. Other studies that used CFA and maximum likelihood estimation to establish the construct validity of the OEQ-II also resulted in moderate model fit (Tieso, 2007b; Siu, 2010; He and Wong, 2014).

### Bayesian structural equation modeling

In contrast to frequentist statistics, which ignores prior knowledge for hypothesis testing, Bayesian statistics relies on Bayes' theorem to update prior knowledge given the data. In maximum likelihood estimation, the parameters of the population are fixed but unknown, and the estimates of those parameters from a sample of the population are random but known. In Bayesian statistics the parameter of the population is considered random, allowing probability statements to be made about its value, as expressed in the prior distribution. Using Bayes' theorem, observed sampling data will revise this prior knowledge, leading to the posterior distribution of the parameter (Bolstad, 2007; Lee, 2007; Kaplan and Depaoli, 2012). Drawing on Bayes theorem, the formula for the posterior distribution *P*(θ|z) of the unknown parameter θ given the observed data z can be expressed as:

$$\begin{array}{l} P\left(\theta |\text{z}\right)=\frac{P\left(\theta ,\text{z}\right)}{P\left(\text{z}\right)}=\frac{P\left(\text{z}|\theta \right)P\left(\theta \right)}{P\left(\text{z}\right)} \end{array}$$

where *P*(θ) stands for the prior distribution of the parameter, reflecting substantive theory or the researcher's prior beliefs, and *P*(z|θ) is referred to as the distribution of the data given the parameter, which represents the likelihood (Levy, 2011; Kaplan and Depaoli, 2012; Kruschke et al., 2012; Zyphur and Oswald, 2015). Omitting the marginal distribution of the data *P*(z) in the formula, reveals the proportionality of the unnormalized posterior distribution to the product of the likelihood and the prior distribution (Levy, 2011; Kaplan and Depaoli, 2012). The uncertainty regarding the population parameter value, as indicated by the variance of its prior probability distribution, is influenced by the observed sampling data, yielding a revised estimate of the parameter, as reflected in its posterior probability distribution (Kaplan and Depaoli, 2012). The Bayesian credibility interval^1^, based on the percentiles of the posterior distribution, allows direct probability statements about the parameter, in contrast to the confidence interval (CI) in frequentist theory, which is contingent on the hypothesis of extensive repeated sampling from the population (Bolstad, 2007; Kaplan and Depaoli, 2012; Zyphur and Oswald, 2015). The posterior distribution *P*(θ|z) yields maximum information about the parameter given the data—“unlike the point estimate and confidence interval in classical statistics, which provide no distributional information” (Kruschke et al., 2012, p. 725). Using a small variance prior, which reflects strong prior knowledge, the data will tend to contribute less information to the construction of the posterior distribution (Muthén and Asparouhov, 2012).

Recently, computational methods (e.g., the Gibbs algorithm) have been developed to draw random samples from the posterior distribution, allowing the practical use of Bayesian statistics (Bolstad, 2007), and leading to strong and increasing interest in this approach to statistics (Kruschke, 2015).

Meanwhile, Muthén and Asparouhov (2012) proposed an innovative approach to factor analysis using Bayesian structural equation modeling (BSEM), which better reflects substantive theory. Many psychological instruments cannot be adequately represented within a frequentist CFA approach, in which each item is allowed to load on one factor and all non-target loadings are fixed at zero (Marsh et al., 2009). Strategies to compensate for this inappropriateness may capitalize on chance (MacCallum et al., 1992), with a large risk of model misspecification (Muthén and Asparouhov, 2013b). In BSEM, parameter specifications of exact zeros are replaced by approximate zeros based on “informative, small-variance priors to reflect the researcher's theories and prior beliefs” (Muthén and Asparouhov, 2012, p. 316).

In the same way, Muthén and Asparouhov (2013a) propose the BSEM approach to measurement invariance analysis across different groups, in which exact zero differences in factor loadings and intercepts are replaced by approximate zero differences based on zero-mean, small-variance priors. “Measurement invariance is built on the notion that a measuring device should function in the same way across varied conditions, so long as those varied conditions are irrelevant to the attribute being measured” (Millsap, 2011, p. 1). With reference to psychological questionnaires, this implies that in order to test for mean differences across groups, the assumption of equivalent measurement of the underlying construct must be fulfilled. Scalar invariance, as characterized by invariant factor loadings and measurement intercepts, is a prerequisite to compare factor means and factor intercepts across groups (Vandenberg and Lance, 2000; Millsap, 2011; Muthén and Asparouhov, 2013c). The BSEM approach to measurement invariance is referred to as approximate measurement invariance and provides a valuable alternative to the multi-group CFA approach to measurement invariance analysis with maximum likelihood estimation (Muthén and Asparouhov, 2013a; Asparouhov and Muthén, 2014), which mostly results in unsatisfactory fit due to minor deviations from exact invariance (Muthén and Asparouhov, 2012). Results of simulation studies indicate that BSEM with approximate measurement invariance is a suitable technique for proper estimation and comparison of factor means and variances across multiple groups that may have non-invariant measurement parameters with minor variance, “without relaxing the invariance specifications or deleting non-invariant items” (Muthén and Asparouhov, 2013a, p. 7; van de Schoot et al., 2013).

### Aim of this study

The first and main objective of this study is to investigate the structural validity of the OEQ-II using BSEM with informative, small-variance priors, and to compare the results of this Bayesian approach to that of a frequentist approach to validation. We hypothesize that maximum likelihood CFA and ESEM models will generate poor data fit by postulating non-estimated parameters as exactly zero. The results of previous validation studies indicate that the OEQ-II—like most psychological instruments—exhibits slight cross-loadings and measures several supplementary minor personality factors in addition to the five overexcitabilities. On the one hand, freeing all cross-loadings and residual covariances leads to a non-identified model (Muthén and Asparouhov, 2012); on the other hand, modifying the model using modification indices in a frequentist analysis may capitalize on chance (MacCallum et al., 1992). Using Bayesian analysis as a pragmatic approach, we hypothesize that the BSEM model will generate a good fit to the data because it may take into account the existence of trivial cross-loadings in the CFA model and many minor correlated residuals among the factor indicators. The BSEM technique allows for the simultaneous inclusion in the model of all, approximate zero cross-loadings and residual covariances based on zero-mean, small-variance priors, and consequently represents substantive theory better.

The second aim of the study is to explore in greater depth the interrelationships between the five overexcitabilities by estimating a higher order model based on theoretical expectations and using Bayesian estimation. Mendaglio and Tillier (2006) strongly advocate the conceptualization of overexcitability within the overall context of development potential in future quantitative studies. According to Dabrowski, an individual's developmental potential is comprised of overexcitability, specific talents and abilities, and a strong autonomous drive to achieve individuality (Dabrowski, 1964, 1972; Mendaglio, 2008). However, the five forms of overexcitability are not equally important with respect to the developmental process (Mendaglio, 2012). Dabrowski considers emotional, intellectual and imaginational overexcitability essential to advanced personality development (Dabrowski, 1972; Mendaglio, 2008, 2012). Positive developmental potential is comprised of all of the five overexcitabilities, although emotional, intellectual and imaginational overexcitability aid the transformation of the lower forms of overexcitability, i.e., psychomotor and sensual overexcitability, “such that their energy is harnessed in the service of the developmental process” (Mendaglio, 2012, p. 212). The exclusive presence of psychomotor and sensual overexcitability constitutes negative developmental potential, which impedes the transcendence of biological needs and societal norms that is considered to be fundamental for the development of autonomy, authenticity, and empathy (Dabrowski, 1972; Mendaglio, 2012). Based on these theoretical considerations we hypothesize that all of the five overexcitabilities will load substantially on a superordinate general construct of positive developmental potential.

The final objective of this study is to investigate approximate invariance of measurement parameters across gender using BSEM. A CFA approach to measurement invariance often proves to be too strict, leading to model rejection and a long series of modifications of the model with a substantial risk of misspecification (Asparouhov and Muthén, 2014). Using an ESEM-Within-CFA approach, the study by Van den Broeck et al. (2014, p. 64) revealed partial strict measurement invariance across gender: “five items showed larger unique variances for girls than for boys, seven thresholds out of 200 were non-invariant, and only 12 out of 250 factor loadings were non-invariant.” Because of the small proportionality of non-invariant parameters, we hypothesize that the BSEM approach will be a useful alternative to establish approximate measurement invariance across gender.

All analyses were carried out using the Mplus software program (Version 7.3; Muthén and Muthén, 1998–2012).

## Materials and methods

### Participants

The OEQ-II (Falk et al., 1999) was added to a study conducted in Flanders investigating the influence of learning patterns on academic performance and the successful transition from secondary to higher education. The self-report measure was translated into Dutch, using back-translation, by the first author of this article, and it was tested on several young adults, in order to ensure the comprehensibility and proper interpretation of the items. The instrument was added to a fifth survey, which was conducted in the first semester of the academic year in which the respondents were in the second consecutive year of a program of higher education. In all, 516 students (318 women: 61.6%; 198 men: 38.4%) completed the OEQ-II online. Of these respondents, 356 (69%) had completed general secondary education before entering higher education, while 26% had followed technical secondary education, 4% had followed vocational secondary education, and 1% had followed secondary education in the arts. Two-thirds of the students were 19 years of age at the time of the survey, while 17% were 18 years old, 10% were 20 years old, and 6% were between 21 and 23 years of age. The study was executed in accordance with the guidelines of the Ethics Committee for the Social Sciences and Humanities of the University of Antwerp with written informed consent from all subjects.

### Instrument

The OEQ-II consists of 50 items, equally representing intellectual overexcitability (e.g., “I love to solve problems and develop new concepts”), imaginational overexcitability (e.g., “Things that I picture in my mind are so vivid that they seem real to me”), emotional overexcitability (e.g., “I am deeply concerned about others”), psychomotor overexcitability (e.g., “If an activity is physically exhausting, I find it satisfying”), and sensual overexcitability (e.g., “I love to listen to the sounds of nature”). The items are scored along a five-point Likert scale with response options ranging from “Not at all like me” to “Very much like me.” A high value on the scale of the items represents a high level of overexcitability.

Significant interrelationships have been found between gender and the extent and nature of overexcitability, as measured by the OEQ-II. A relatively strong association of emotional and sensual overexcitability with the female gender appears to be a general empirical finding (Bouchet and Falk, 2001; Treat, 2006; Tieso, 2007a,b; Miller et al., 2009; Siu, 2010; Wirthwein et al., 2011; He and Wong, 2014; Van den Broeck et al., 2014; Botella et al., 2015). There is also evidence of a stronger relationship between the dispositional traits of intellectual (Bouchet and Falk, 2001; Treat, 2006; Miller et al., 2009; Rinn et al., 2010; Siu, 2010; Van den Broeck et al., 2014; Botella et al., 2015) and psychomotor (Bouchet and Falk, 2001; Treat, 2006; Rinn et al., 2010; He and Wong, 2014; Van den Broeck et al., 2014; Botella et al., 2015) overexcitability and the male gender. Because of these interrelationships, statistical analyses will be performed for the different gender groups separately.

### MCMC convergence

Bayesian estimation makes use of Markov chain Monte Carlo (MCMC) algorithms to iteratively draw random samples from the posterior distribution of the model parameters (Muthén and Muthén, 1998–2012). The software program Mplus uses the Gibbs algorithm (Geman and Geman, 1984) to execute MCMC sampling. MCMC convergence of posterior parameters, which indicates that a sufficient number of samples has been drawn from the posterior distribution to accurately estimate the posterior parameter values (Arbuckle, 2013), is evaluated via the potential scale reduction (PSR) convergence criterion (Gelman and Rubin, 1992; Gelman et al., 2014). The PSR criterion compares within- and between-chain variation of parameter estimates. When a single MCMC chain is used, the PSR compares variation within and between the third and fourth quarters of the iterations. A PSR value of 1.000 represents perfect convergence (Muthén and Muthén, 1998–2012; Kaplan and Depaoli, 2012). With a large number of parameters, a PSR < 1.100 for each parameter indicates that convergence of the MCMC sequence has been obtained (Muthén and Muthén, 1998–2012).

Convergence of the MCMC algorithm in distribution is assessed via monitoring of the posterior distributions by trace and autocorrelation plots (Muthén and Muthén, 1998–2012). Posterior parameter trace plots display the sampled parameter values over time. Quick up-and-down fluctuations and absence of long-term trends in the plot indicate rapid convergence in distribution (Kaplan and Depaoli, 2012; Arbuckle, 2013).

Autocorrelation plots also display the degree of non-independence of successive posterior draws in the MCMC chains Muthén and Muthén, 1998–2012; Kaplan and Depaoli, 2012). An estimated correlation between the sampled parameter values reaching zero indicates convergence (Arbuckle, 2013).

### Analyses

Before performing a Bayesian analysis of the OEQ-II model, a maximum likelihood analysis was carried out for comparison purposes. Using maximum likelihood estimation, a CFA model was tested—according to the OEQ-II's hypothesized latent factor loading pattern for the 50 observed variables—and an exploratory factor analysis (EFA) for five factors was performed using ESEM with oblique Geomin rotation. In the ESEM model, the five correlated factors were measured by each of the 50 factor indicators and the residuals were not correlated.

Subsequently, a Bayesian analysis was performed using the CFA model, albeit with informative, small-variance priors for the cross-loadings in the model and uncorrelated residuals. Target loadings with non-informative priors—i.e., normally distributed priors with a mean of zero and infinite variance—and cross-loadings with strong informative priors—i.e., normally distributed priors with a mean of zero and a variance of 0.01, yielding 95% small cross-loading bounds of ±0.20 (Muthén and Asparouhov, 2012)—were utilized in this model. Applying the Bayes estimator and Gibbs algorithm, two independent MCMC chains with 10,000 iterations were used to describe the posterior distribution. The factor variances were fixed at one to set the metric of the factors, and standardized variables were analyzed.

In the next step, a Bayesian analysis was performed using informative, small-variance priors for cross-loadings and residual covariances in the CFA model. In this BSEM analysis, normal prior distributions *N*(0, 0.01) were used for the cross-loadings, admitting ignorable effect sizes (Muthén and Asparouhov, 2012). An inverse-Wishart prior distribution *IW*(0, *df*) with *df* = 56 was applied for the correlated residuals, corresponding to prior zero-means and variances of 0.01 (MacKinnon, 2008). In this analysis, every 10th iteration was used—in order to reduce autocorrelation between successive posterior draws—with a total of 100,000 iterations and one MCMC chain to describe the posterior distribution. A sensitivity analysis was carried out, in which the effect of varying the prior variances of the residual covariances on the parameter estimates and model fit was investigated.

**Figure 1:**
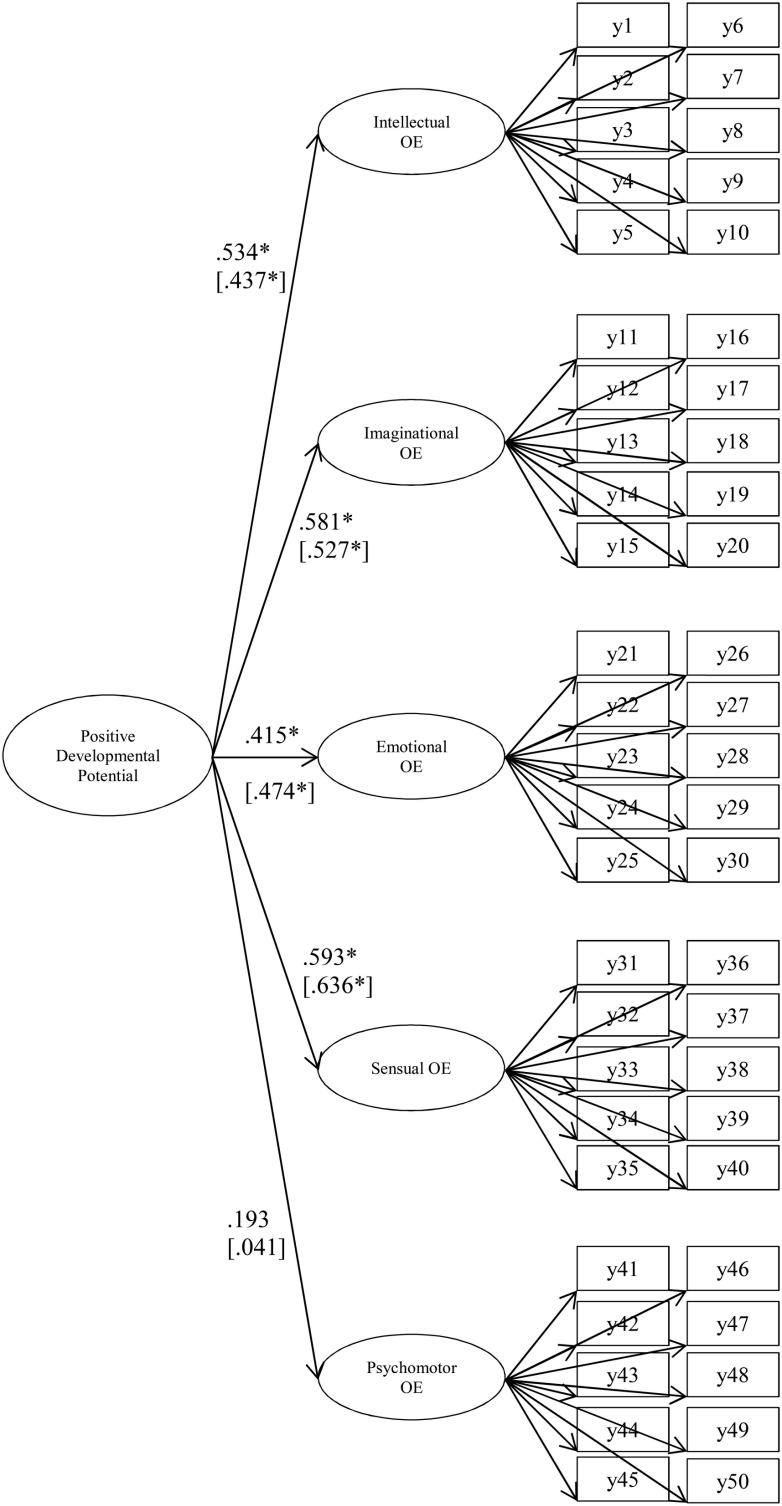
**Higher order BSEM model with informative, small-variance priors for cross-loadings and residual covariances for the Overexcitability Questionnaire-Two (OEQ-II; Falk et al., 1999) data for females and males (second-order factor loadings are added within parentheses)**. OE, overexcitability; BSEM, Bayesian structural equation modeling. ^*^Significance at the 5% level in the sense that the 95% Bayesian credibility interval does not cover zero.

In relation to the second aim of this study, a higher order model was estimated—according to the hypothesized latent factor loading pattern, as represented in Figure 1—using BSEM with informative, small-variance priors for the cross-loadings λ ~ *N*(0, 0.01) and residual covariances δ ~ *IW*(0, 56) in the measurement model. In the hypothetical model, the latent variable of positive developmental potential was operationalized according to the five overexcitability factors.

Finally, a Bayesian multiple-group model with approximate measurement invariance (Muthén and Asparouhov, 2013a) was carried out. One categorical latent variable with two known classes (i.e., male and female) was specified in this BSEM model. Normally distributed priors *N*(0, 0.01) were utilized for differences in intercepts and factor loadings across groups. Non-informative or diffuse priors were used for factor means, variances, and covariances across groups with the exception of factor means and variances in the male group, which were set at zero and one, respectively. Residuals were correlated, using an inverse-Wishart prior distribution *IW*(0, 16), corresponding to prior zero-means and variances of 0.01. Analyses were executed for each overexcitability factor and the alignment optimization method with Bayes estimation (Asparouhov and Muthén, 2014) was applied, which optimizes alignment “of the measurement parameters, factor loadings and intercepts/thresholds according to a simplicity criterion that favors few non-invariant measurement parameters,” and subsequently adjusts “the factor means and variances in line with the optimal alignment” (Muthén and Muthén, 2013, p. 2). The alignment optimization method provides a solution to a parameterization indeterminacy, in which (few) non-invariant parameters are underestimated and (many) invariant parameters are overestimated, due to non-normally distributed deviations from a parameter average over groups resulting in the misestimation of factor means and variances (Muthén and Asparouhov, 2013a). In this BSEM multiple-group analysis, every 10th iteration was saved with a maximum and minimum number of iterations for each of two MCMC chains of 50,000 and 1,000, respectively, using the Gelman-Rubin PSR < 1.05 criterion (Gelman and Rubin, 1992).

### Model fit assessment

The following fit measures were used as a means of evaluating the quality of the fit of both CFA and EFA models: the chi-square statistic, comparative fit index (CFI; Bentler, 1990), and root mean square error of approximation (RMSEA; Steiger, 1990). A non-significant chi-square value, CFI values close to 1 (Hu and Bentler, 1995), and a value of the RMSEA of 0.05 or less (Browne and Cudeck, 1989) indicate a close fit of the model.

For the BSEM models, fit assessment was carried out using Posterior Predictive Checking in which—as implemented in Mplus—the likelihood-ratio chi-square statistic for the observed data is compared to the chi-square based on synthetic data obtained by means of draws of parameter values from the posterior distribution (Muthén and Muthén, 1998–2012; Scheines et al., 1999; Asparouhov and Muthén, 2010). The simulated data should approximately match the observed data if the model fits the data (Kaplan and Depaoli, 2012). The Posterior Predictive *p*-value (PP*p*) measures the proportion of the chi-square values of the replicated data that exceeds that of the observed data. A low PP*p* (<0.05) indicates poor model fit. On the contrary, a PP*p* of 0.50, as well as a 95% CI for the difference in the chi-square statistic for the observed and simulated data that contains zero positioned close to the middle of the interval, are both indicative of excellent model fit (Muthén and Asparouhov, 2012). Results of simulation studies show the PP*p* to demonstrate sufficient power to reveal important model misspecifications (Muthén and Asparouhov, 2012).

## Results

### Descriptive statistics

Descriptive summary statistics for the five overexcitability factors are reported per gender group in Table 1. The mean outcomes are consistent with all other studies using the OEQ-II, in which the two highest scores have been for emotional, intellectual, or psychomotor overexcitability (Falk and Miller, 2009).

**Table 1:** **Descriptive statistics per overexcitability factor for females and males**.

	**Females**					**Males**				
	**IOE**	**ImOE**	**EOE**	**SOE**	**POE**	**IOE**	**ImOE**	**EOE**	**SOE**	**POE**
Mean	3.450	2.809	3.737	3.295	3.233	3.540	2.708	3.162	3.112	3.380
Standard deviation	0.591	0.779	0.572	0.736	0.714	0.538	0.663	0.617	0.691	0.700
Skewness	−0.035	0.220	−0.245	−0.147	0.105	0.161	0.148	−0.097	0.041	−0.253
Kurtosis	0.102	−0.195	−0.153	−0.175	−0.217	−0.128	−0.245	0.148	0.054	−0.094

*IOE, intellectual overexcitability; ImOE, imaginational overexcitability; EOE, emotional overexcitability; SOE, sensual overexcitability; POE, psychomotor overexcitability*.

### Confirmatory and exploratory factor analysis

Table 2 shows the chi-square statistic, CFI, and RMSEA for the evaluation of both maximum likelihood CFA and EFA models. Highly significant chi-square statistics, RMSEA values of more than 0.05, and CFI values of less than 0.90 all indicate that the CFA and EFA models for females and males fit the data poorly. Moreover, as represented in Table 3, the hypothesized five factor pattern is not fully recovered by the EFA results for females and males. Several significant (at the 5% significance level) cross-loadings on other latent factors can be detected. The hypothesized factor loading pattern is not well captured by the EFA model, possibly due to the existence of many minor correlated residuals among the factor indicators (Muthén and Asparouhov, 2012), as can be expected from inspection of the modification indices.

**Table 2:** **Maximum likelihood CFA and EFA model testing results for females (***n*** = 318) and males (***n*** = 198)**.

**Model**	**χ^2^**	***df***	***p*-value**	**RMSEA**	**CFI**
**FEMALES**					
CFA	2565	1165	0.000	0.061	0.767
EFA	1934	985	0.000	0.055	0.842
**MALES**					
CFA	2174	1165	0.000	0.066	0.712
EFA	1660	985	0.000	0.059	0.807

*CFA, confirmatory factor analysis; EFA, exploratory factor analysis; df, degrees of freedom; RMSEA, root mean square error of approximation; CFI, comparative fit index*.

**Table 3:** **Maximum likelihood EFA model estimation results for females (***n*** = 318) and males (***n*** = 198)**.

	**Females**					**Males**				
	**F1**	**F2**	**F3**	**F4**	**F5**	**F1**	**F2**	**F3**	**F4**	**F5**
**FACTOR LOADINGS**										
y1	0.467^*^	0.086	−0.12	−0.018	−0.058	0.405^*^	0.163	0.101	−0.021	−0.034
y2	0.011	0.135^*^	0.041	0.03	0.023	−0.042	0.263^*^	−0.041	0.079	0.032
y3	0.498^*^	−0.031	0.117	0.153^*^	−0.024	0.550^*^	−0.041	0.026	−0.017	−0.123
y4	0.599^*^	0.038	0.029	−0.055	0.018	0.476^*^	−0.08	0.027	0.221^*^	−0.064
y5	0.504^*^	0.044	0.016	0.146^*^	0.003	0.443^*^	−0.013	0.133	0.021	−0.170^*^
y6	0.589^*^	0.073	0.061	−0.06	−0.007	0.425^*^	0.251^*^	0.005	0.042	−0.113
y7	0.667^*^	−0.115^*^	−0.1	−0.002	0.126^*^	0.652^*^	−0.023	−0.101	0.07	0.088
y8	0.610^*^	−0.003	0.162^*^	0.113^*^	−0.056	0.607^*^	−0.012	0.145^*^	0.068	−0.069
y9	0.575^*^	−0.004	0.034	0.169^*^	0.013	0.558^*^	0.079	0.121	−0.142	0.058
y10	0.685^*^	−0.031	0.102	−0.044	0.014	0.694^*^	0.05	−0.001	0.112	0.025
y11	0.008	0.480^*^	0.084	0.075	−0.026	0.361^*^	0.291^*^	−0.01	−0.047	−0.045
y12	0.006	0.631^*^	0.11	0.011	0.051	0.223^*^	0.429^*^	0.025	0.144	−0.147^*^
y13	−0.052	0.466^*^	0.173^*^	0.059	−0.085	0.254^*^	0.382^*^	0.037	−0.048	0.058
y14	0.037	0.569^*^	−0.058	0.04	−0.076	−0.041	0.582^*^	0.023	−0.072	0.066
y15	0.029	0.664^*^	0.074	−0.068	0.099^*^	0.005	0.442^*^	0.001	0.157	−0.084
y16	−0.011	0.725^*^	0.01	−0.027	0.026	0.084	0.744^*^	−0.039	−0.017	0.029
y17	0.062	0.570^*^	−0.048	0.044	0.038	0.082	0.563^*^	−0.11	−0.019	0.171^*^
y18	0	0.504^*^	0.119	−0.055	0.032	−0.252^*^	0.506^*^	0.002	0.272^*^	−0.037
y19	0.045	0.546^*^	0.212^*^	−0.029	0.101^*^	−0.123	0.455^*^	0.026	0.425^*^	0.017
y20	0.076	0.410^*^	0.353^*^	−0.014	−0.082	0.180^*^	0.354^*^	−0.022	0.255^*^	−0.117
y21	0.236^*^	−0.012	0.041	0.577^*^	0.038	0.104	−0.082	0.571^*^	−0.059	−0.001
y22	−0.132	0.303^*^	−0.106	0.402^*^	−0.007	0.061	−0.01	0.492^*^	−0.046	0.001
y23	0.036	−0.023	0.01	0.477^*^	0.125^*^	−0.007	−0.024	0.571^*^	−0.058	0.078
y24	0.172^*^	0.147^*^	−0.038	0.185^*^	0.184^*^	0.025	0.270^*^	0.079	0.248^*^	0.140^*^
y25	0.012	0.537^*^	−0.140^*^	0.412^*^	−0.068	0.101	0.359^*^	0.389^*^	0.012	−0.012
y26	0.143^*^	0.008	0.051	0.605^*^	0.051	0.021	0.005	0.847^*^	0.029	0.006
y27	−0.02	0.406^*^	0.027	0.468^*^	0.062	−0.175^*^	0.471^*^	0.311^*^	0.118	−0.074
y28	0.240^*^	0.323^*^	−0.052	0.486^*^	−0.061	0.179^*^	0.039	0.351^*^	0.082	0.028
y29	−0.101	−0.084	0.210^*^	0.633^*^	0.009	0.038	−0.105	0.507^*^	0.041	−0.016
y30	0.126	0.138^*^	0.05	0.347^*^	0.031	−0.04	0.11	0.462^*^	0.220^*^	0.045
y31	0.188^*^	0.025	0.465^*^	−0.071	0.188^*^	0.308^*^	−0.076	−0.001	0.465^*^	−0.007
y32	0.134^*^	0.037	0.583^*^	−0.132^*^	−0.025	0.068	0.015	0.046	0.644^*^	−0.067
y33	0.189^*^	0.004	0.555^*^	−0.088	−0.018	−0.071	0.072	0.127^*^	0.756^*^	−0.058
y34	−0.056	0.208^*^	0.264^*^	0.114	0.111^*^	0.133	0.094	0.09	0.179^*^	0.084
y35	−0.038	0.221^*^	0.657^*^	−0.04	0.005	0.003	0.325^*^	−0.054	0.543^*^	0.008
y36	0.252^*^	0.032	0.472^*^	0.113^*^	0.028	0.185^*^	0.061	0.227^*^	0.420^*^	0.107
y37	−0.023	−0.073	0.741^*^	0.141^*^	−0.021	0.153	−0.028	0.033	0.462^*^	0.042
y38	0	0.026	0.834^*^	0.062	−0.001	0.184^*^	−0.043	−0.029	0.794^*^	0.008
y39	0.056	0.030	0.692^*^	0.029	−0.036	0.233^*^	0.091	−0.061	0.453^*^	0.11
y40	0.184^*^	0.033	0.495^*^	0.03	0.027	−0.002	0.037	0.189^*^	0.392^*^	0.124
y41	0.282^*^	0.058	−0.295^*^	−0.019	0.289^*^	0.255^*^	−0.097	−0.014	−0.093	0.342^*^
y42	0.007	−0.072	−0.058	0.093^*^	0.753^*^	0.145^*^	−0.082	−0.033	0.036	0.799^*^
y43	−0.019	−0.104^*^	0.001	0.041	0.832^*^	−0.012	−0.112	−0.013	0.118^*^	0.871^*^
y44	−0.057	−0.034	0.003	0.058	0.751^*^	0.009	−0.007	0.127^*^	−0.029	0.789^*^
y45	0.098	0.065	−0.045	−0.181^*^	0.614^*^	−0.072	−0.007	0.009	0.087	0.594^*^
y46	−0.032	0.195^*^	0.042	−0.016	0.419^*^	−0.083	0.180^*^	0.155	−0.094	0.353^*^
y47	−0.073	0.275^*^	0.114^*^	−0.064	0.503^*^	−0.131	0.293^*^	0.029	0.023	0.579^*^
y48	−0.049	0.063	−0.041	0.149^*^	0.612^*^	−0.009	0.136	0.131	−0.177^*^	0.514^*^
y49	0.073	−0.097	0.053	0.013	0.691^*^	−0.04	−0.037	0.215^*^	0.076	0.408^*^
y50	0.086	0.058	−0.002	−0.036	0.707^*^	0.066	0.04	−0.071	−0.150^*^	0.653^*^
**FACTOR CORRELATIONS**										
F1	1.000					1.000				
F2	0.292^*^	1.000				0.036	1.000			
F3	0.345^*^	0.325^*^	1.000			0.226^*^	0.228^*^	1.000		
F4	0.082	0.057	0.083	1.000		0.252^*^	0.344^*^	0.252^*^	1.000	
F5	0.133^*^	0.114^*^	0.010	0.126^*^	1.000	−0.040	−0.056	0.139	−0.038	1.000

*EFA, exploratory factor analysis; F1, factor 1; F2, factor 2; F3, factor 3; F4, factor 4; F5, factor 5. The standardized coefficients in bold represent factor loadings that are the largest for each factor indicator*.

* *p < 0.05*.

### BSEM with informative, small-variance priors for cross-loadings

Table 4 presents the fit results of the BSEM model with informative, small-variance priors for cross-loadings for both gender groups. The 95% CIs for the difference between the observed and replicated chi-square values do not cover zero, and the PP*p*s are smaller than 0.05, both indicating unsatisfactory model fit. The results of this BSEM model, which are not reported, still reveal significant^2^ (in the sense that the 95% Bayesian credibility interval does not encompass zero) but fewer cross-loadings and slightly higher major factor loadings, as compared with the EFA model. Increasing the variance of the prior distributions of the cross-loadings does not alter the fit results considerably. We may assume that the OEQ-II measures several supplementary minor personality factors in addition to the five overexcitabilities. On the one hand, freeing all residual covariances would lead to a non-identified model (Muthén and Asparouhov, 2012), which in Bayesian analysis may hinder MCMC convergence; on the other hand, modifying the model using modification indices in a frequentist analysis may capitalize on chance (MacCallum et al., 1992), with a large risk of model misspecification (Muthén and Asparouhov, 2013b).

**Table 4:** **Bayesian model testing results for females (***n*** = 318) and males (***n*** = 198)**.

**Model**	**PP *p***	**95% CI**
**FEMALES**		
CFA with cross-loadings	0.000	770.367−994.541
CFA with cross-loadings and residual covariances	0.767	−199.724–93.706
**MALES**		
CFA with cross-loadings	0.000	448.610–682.850
CFA with cross-loadings and residual covariances	0.905	−248.311–50.020

*PP p, posterior predictive probability; CI, confidence interval; CFA, confirmatory factor analysis*.

### BSEM with informative, small-variance priors for cross-loadings and residual covariances

**Figure 2:**
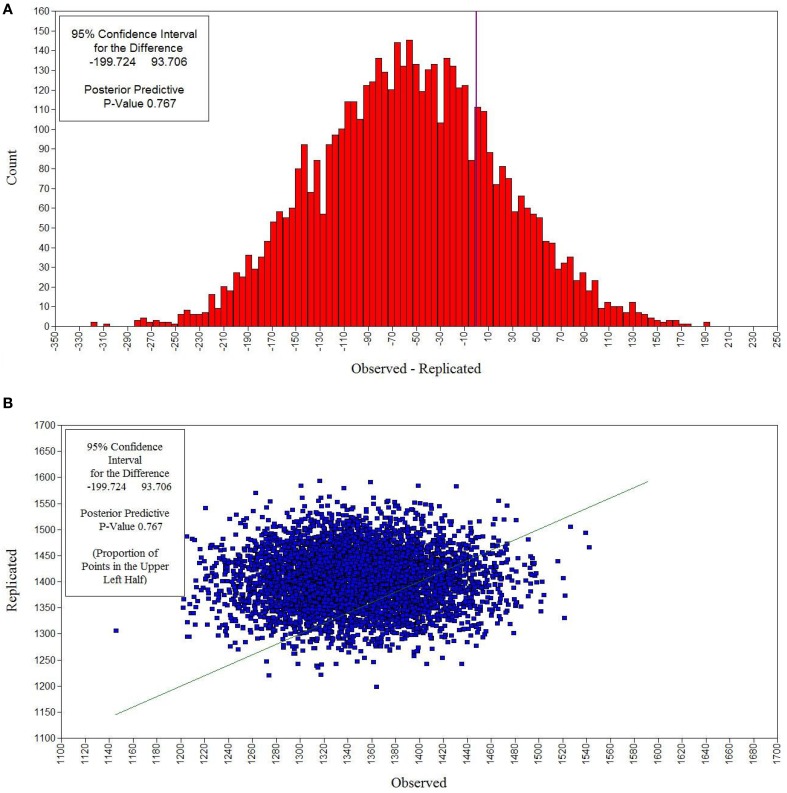
**Bayesian posterior predictive checking distribution plot (A) and scatterplot (B) for the Bayesian model with small-variance priors for cross-loadings and residual covariances for females**. In the posterior predictive checking distribution plot, the chi-square statistic for the observed data is marked by the vertical line, which corresponds to a zero value on the *x*-axis. The matching scatterplot allows determining the PP*p* as the proportion of points above the 45 degree line.

Subsequently, a Bayesian analysis was performed using informative, small-variance priors for cross-loadings and residual covariances. As represented in Table 4, the 95% CIs for the difference between the observed and the replicated chi-square values cover zero and the PP*p*s are 0.767 and 0.905 for the female and male group, respectively, both indicating good model fit. Figure 2A presents the distribution of the difference between the observed and the replicated chi-square values for the female group. The matching scatterplot (see Figure 2B), with the majority of the points plotted along the 45 degree line, indicates satisfactory model fit for the observed data.

**Figure 3:**
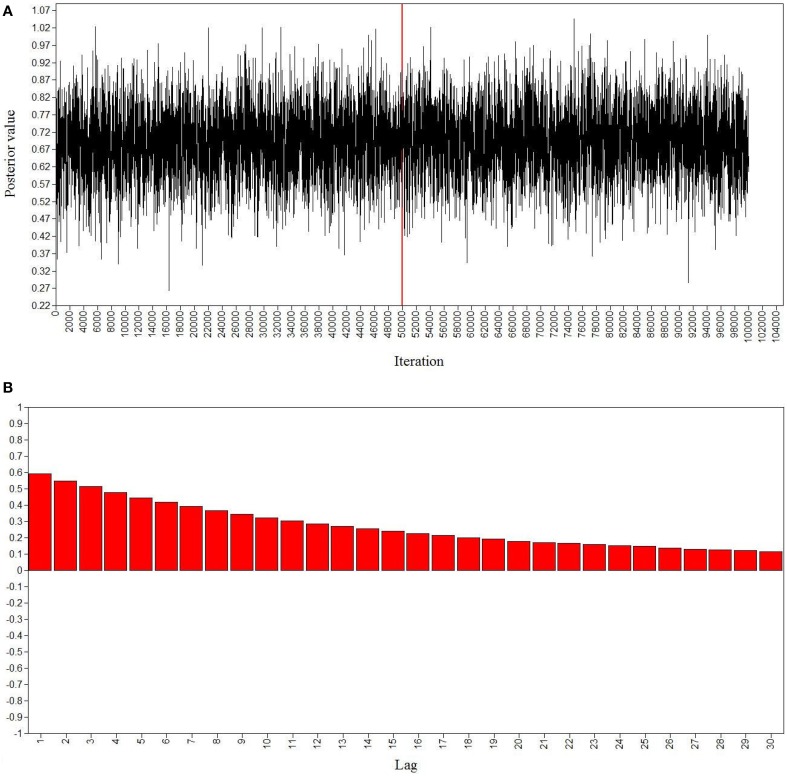
**Bayesian posterior parameter trace plot (A) and autocorrelation plot (B) for the loading of item y10 on intellectual overexcitability in the Bayesian model with small-variance priors for cross-loadings and residual covariances for males**. The *x*-axis of the posterior parameter trace plot displays the iterations of the MCMC procedures and the *y*-axis shows the corresponding parameter values. The vertical line represents the burn-in phase at 50,000 iterations. The iterations on the right-hand side of the vertical line determine the posterior distribution of the loading estimate.

Good MCMC convergence was established for the two models. The PSR value smoothly decreased over the iterations, reaching a value of 1.010 after half of the iterations. Additionally, the stability of the parameter estimates across the iterations was verified. Figure 3 presents posterior parameter trace and autocorrelation plots for the loading of item y10 on the intellectual overexcitability factor for the male group. The trace plot (see Figure 3A) displays a stable, horizontal band for the parameter presented, indicating convergence of the MCMC algorithm in distribution. The autocorrelation plot (see Figure 3B) displays low autocorrelation or approximate non-independence of successive posterior samples. The posterior parameter trace and autocorrelation plots for the other parameters included in the models (not reported) were also indicative of good convergence in distribution.

Thus, the results of both BSEM models can be reliably interpreted. With the exception of one non-significant (in the sense that the 95% Bayesian credibility interval encompasses zero) major factor loading, the hypothesized factor loading pattern was fully recovered, with substantial target loadings and only one significant cross-loading (in the male group), as displayed in Table 5 (in Mplus, the reported estimates are the medians of their posterior distributions). Many minor residual covariances were found to be significant at the 5% level, particularly 49 (i.e., 4%) for the female group, with an average absolute residual correlation (range) of 0.221 (−0.254 to 0.532), and 68 (i.e., 5.55%) for the male group, with an average absolute residual correlation (range) of 0.241 (−0.294 to 0.462). Excluding these residual correlations may lead to the poor fit of the previously studied models (Cole et al., 2007), and unsatisfactory loading pattern recovery in the ESEM model (Muthén and Asparouhov, 2012). The Bayesian factor correlations are located in order of magnitude between the maximum likelihood EFA (smallest values, cf. Tables 3, 5) and CFA (largest values, not reported) correlations. However, according to theory, the factors are predicted to correlate to a considerable level. Table 5 shows weak to moderate factor correlations.

**Table 5:** **Bayesian model estimation results for females (***n*** = 318) and males (***n*** = 198) using small-variance priors for cross-loadings and residual covariances**.

	**Females**					**Males**				
	**IOE**	**ImOE**	**EOE**	**SOE**	**POE**	**IOE**	**ImOE**	**EOE**	**SOE**	**POE**
**FACTOR LOADINGS**										
y1	0.456^*^	0.025	−0.004	−0.042	−0.035	0.530^*^	0.069	0.028	−0.041	0.013
y2	**0.078**	0.06	0.046	0.007	0.005	**0.178**	0.021	−0.011	0.024	−0.003
y3	0.587^*^	0.015	0.031	0.039	−0.013	0.611^*^	−0.004	−0.019	−0.047	−0.047
y4	0.576^*^	0.001	0.023	0.031	0.003	0.615^*^	−0.034	−0.001	0.041	−0.014
y5	0.634^*^	−0.021	0.051	−0.016	0.008	0.617^*^	−0.05	0.035	−0.018	−0.085
y6	0.655^*^	0.035	−0.07	0.042	−0.007	0.555^*^	0.061	0.003	0.031	−0.06
y7	0.689^*^	−0.04	−0.028	−0.121	0.073	0.728^*^	−0.025	−0.103	−0.016	0.076
y8	0.619^*^	0.028	0.044	0.099	−0.054	0.680^*^	−0.03	0.06	0.041	−0.03
y9	0.649^*^	−0.033	0.07	0.002	0.011	0.574^*^	0.022	0.039	−0.039	0.074
y10	0.703^*^	0.026	−0.038	0.024	−0.01	0.663^*^	0.048	−0.006	0.068	0.028
y11	−0.03	0.688^*^	−0.002	−0.04	0.008	0.032	0.563^*^	0.018	0.004	−0.031
y12	−0.002	0.729^*^	0.012	−0.022	0.02	0.014	0.681^*^	0.011	0.035	−0.082
y13	−0.042	0.684^*^	−0.015	0.003	−0.058	−0.003	0.648^*^	−0.013	−0.024	0.046
y14	−0.001	0.488^*^	0.04	−0.016	−0.034	0.006	0.404^*^	0.047	−0.048	0.031
y15	0.027	0.681^*^	−0.005	−0.036	0.085	0.024	0.475^*^	−0.015	0.009	−0.017
y16	0.009	0.688^*^	0.001	−0.045	0	0.044	0.666^*^	0.008	−0.092	0.026
y17	0.019	0.505^*^	0.044	−0.003	0.026	−0.02	0.563^*^	−0.033	−0.058	0.112
y18	−0.021	0.497^*^	0.016	0.06	0.006	−0.075	0.424^*^	0.017	0.042	−0.017
y19	0.069	0.513^*^	0.024	0.112	0.043	0.017	0.404^*^	0.034	0.191^*^	−0.001
y20	0.039	0.608^*^	−0.007	0.14	−0.077	0.001	0.607^*^	0.009	0.08	−0.092
y21	0.071	−0.047	0.650^*^	0.006	0.009	0.011	0.006	0.565^*^	−0.066	0.045
y22	−0.067	0.042	0.422^*^	−0.04	0.008	−0.017	−0.053	0.652^*^	−0.052	−0.033
y23	−0.054	−0.033	0.540^*^	−0.021	0.056	−0.018	−0.05	0.584^*^	−0.041	0.036
y24	0.083	0.029	0.347^*^	−0.029	0.109	0.038	0.051	0.359^*^	0.062	0.076
y25	−0.022	0.126	0.567^*^	−0.031	−0.058	0.031	0.049	0.629^*^	0.039	−0.031
y26	0.003	−0.059	0.723^*^	−0.011	−0.01	0.057	−0.011	0.750^*^	−0.017	0.045
y27	−0.027	0.092	0.628^*^	−0.005	0.036	−0.088	0.121	0.588^*^	−0.013	−0.075
y28	0.098	0.066	0.596^*^	0.027	−0.034	0.029	0.035	0.474^*^	0.076	0.019
y29	−0.067	−0.122	0.640^*^	0.044	−0.026	−0.018	−0.073	0.572^*^	0.035	−0.004
y30	0.059	0.007	0.457^*^	0.026	0.014	0.012	0.005	0.546^*^	0.085	−0.004
y31	0.049	−0.005	−0.051	0.614^*^	0.125	0.009	−0.045	−0.041	0.709^*^	−0.035
y32	0.014	−0.02	−0.071	0.692^*^	−0.018	0.02	0.025	−0.043	0.671^*^	−0.025
y33	0.045	−0.01	−0.026	0.648^*^	−0.022	−0.015	0.01	0.056	0.679^*^	−0.013
y34	−0.094	0.026	0.087	0.444^*^	0.06	0.044	−0.014	0.049	0.363^*^	0.016
y35	−0.042	0.09	−0.006	0.693^*^	−0.02	0.015	0.106	0.003	0.540^*^	0.014
y36	0.091	0.009	0.042	0.623^*^	0.02	0.029	0.036	0.084	0.604^*^	0.076
y37	−0.033	−0.022	0.042	0.694^*^	−0.038	−0.017	−0.064	−0.009	0.679^*^	0.005
y38	−0.031	0.004	0.041	0.790^*^	−0.025	0.038	−0.017	−0.004	0.787^*^	−0.039
y39	0.012	−0.004	−0.022	0.732^*^	−0.03	0.023	0.055	−0.03	0.591^*^	0.048
y40	0.032	0.024	−0.009	0.622^*^	0.025	−0.044	0.032	0.066	0.491^*^	0.041
y41	0.091	−0.017	0.006	−0.062	0.356^*^	0.091	−0.047	0.013	−0.023	0.421^*^
y42	−0.045	−0.047	0.043	−0.004	0.729^*^	0.017	0.019	−0.04	0.034	0.758^*^
y43	−0.013	−0.063	−0.008	−0.008	0.796^*^	−0.036	−0.041	−0.016	0.046	0.802^*^
y44	−0.034	0.049	−0.029	−0.034	0.761^*^	−0.016	−0.009	0.093	−0.03	0.752^*^
y45	0.02	0.038	−0.076	−0.021	0.673^*^	−0.024	0.014	−0.058	0.023	0.700^*^
y46	0.006	0.075	0.02	0.022	0.524^*^	−0.038	0.042	0.028	−0.007	0.529^*^
y47	−0.046	0.099	0.02	0.105	0.564^*^	0.016	0.021	−0.01	0.029	0.675^*^
y48	−0.003	0.000	0.058	−0.048	0.707^*^	−0.019	0.01	0.022	−0.02	0.634^*^
y49	0.048	−0.103	0.001	0.05	0.730^*^	0.045	−0.039	0.1	−0.011	0.542^*^
y50	0.014	0.029	0.000	0.029	0.716^*^	−0.019	−0.01	−0.063	−0.036	0.670^*^
**FACTOR CORRELATIONS**										
IOE	1.000					1.000				
ImOE	0.343^*^	1.000				0.334^*^	1.000			
EOE	0.336^*^	0.368^*^	1.000			0.318^*^	0.367^*^	1.000		
SOE	0.471^*^	0.506^*^	0.288^*^	1.000		0.462^*^	0.476^*^	0.426^*^	1.000	
POE	0.163	0.144	0.215^*^	0.071	1.000	−0.042	−0.022	0.166	0.035	1.000

*IOE, intellectual overexcitability; ImOE, imaginational overexcitability; EOE, emotional overexcitability; SOE, sensual overexcitability; POE, psychomotor overexcitability. The standardized coefficients in bold represent factor loadings that are the largest for each factor indicator*.

* *Significance at the 5% level in the sense that the 95% Bayesian credibility interval does not cover zero*.

A sensitivity analysis was carried out, investigating the effects of varying the prior variances of the residual covariances on the PP*p* and the lower and upper bounds of the 95% CI for the difference in chi-square statistic for the observed and synthetic data. This analysis also checked the variability of the parameter estimates. Unless the research sample is extremely small, or the model and/or prior distribution are strongly contradicted by the data, the results of the Bayesian analysis will change very little when the variance of the prior distribution is altered (Arbuckle, 2013). Table 6 presents the Bayesian model fit results under varying prior variance conditions for the male group, and also presents the standardized estimate of the factor loading of item y1 on the latent variable of intellectual overexcitability. Initially, an inverse-Wishart prior *IW*(0, 56) was used for the residual covariances, corresponding to prior zero-means and variances of 0.0111 (*SD* = 0.1054). Augmenting the degrees of freedom for the parameters that are assumed to follow an inverse-Wishart distribution will decrease the variance of the prior distribution or increase the degree of prior knowledge included in the model. The extent to which the prior variance can be reduced is monitored through the PP*p*. In the framework of this residual correlations sensitivity analysis, both a less informative prior with *df* = 54 (corresponding to a prior variance of 0.0833) and more informative priors with *df* = 66, 76, and 86 (corresponding to prior variances of 0.0003, 0.0001, and < 0.0001, respectively) were used. Applying a strong informative prior with *df* = 73 (corresponding to a prior variance of 0.0001) yielded excellent model fit, as indicated by a PP*p* of 0.515. However, for both gender groups, the results of the sensitivity analysis indicate that different priors for the residual covariances do not alter the estimation of the factor loadings considerably. Additionally, with rather large sample sizes, the choice of the prior variance is less important as the data contribute more information to the construction of the posterior distribution (Muthén and Asparouhov, 2012).

**Table 6:** **Bayesian model testing results for males using small-variance priors for cross-loadings and varying prior variance conditions for residual covariances, and corresponding estimation results for the factor loading of item y1 on intellectual overexcitability**.

**Model**			**Parameter**			**95% Credibility interval**	
***df***	**PP *p***	**95% CI**	**Loading λ*_1_***	**Posterior *SD***	**One-tailed *p***	**Lower 2.5%**	**Upper 2.5%**
54	0.914	−256.198–44.962	0.526	0.102	0.000	0.314	0.712
56	0.905	−248.311–50.020	0.530	0.104	0.000	0.312	0.716
66	0.728	−204.914–101.821	0.537	0.099	0.000	0.330	0.718
73	0.515	−163.316–153.099	0.539	0.096	0.000	0.340	0.715
76	0.414	−132.449–171.688	0.536	0.098	0.000	0.334	0.719
86	0.108	−60.272–257.655	0.544	0.094	0.000	0.347	0.716

*df, degrees of freedom; PP p, posterior predictive probability; CI, confidence interval; SD, standard deviation*.

### BSEM higher order model

With respect to the female group, the 95% CI for the difference between the observed and the replicated chi-square values covers zero, with a lower bound of -197.241 and an upper bound of 92.474, and the PP*p* is 0.757, both indicating good model fit. The same conclusion can be drawn for the male group (PP*p* = 0.884, Δ observed and replicated χ^2^ 95% CI [−246.146, 59.556]). A steadily decreasing PSR value, with a value close to 1 for the last few tens of thousands of iterations, as well as convergence plots showing tight horizontal bands for the parameters, and autocorrelation plots displaying low dependence in the chain, are all indicative of good MCMC convergence.

The hypothesized loading pattern depicted in Figure 1 is only partially recovered for both gender groups. Psychomotor overexcitability can be distinguished from the other forms of overexcitability, as indicated by the non-significant factor loading on the general latent construct of positive developmental potential. Regarding the measurement model, all intended factor loadings—with the exception of the loading of item y2 on intellectual overexcitability, as in the previous BSEM models—were substantive. Nonetheless, some cross-loadings were found to deviate substantially from zero, particularly 6 for the female group, with an average loading of 0.175, and 2 for the male group, with an average loading of 0.224. Many minor residual covariances were found to be significant at the 5% level, particularly 34 for the female group, with an average absolute residual correlation (range) of 0.233 (−0.252 to 0.532), and 47 for the male group, with an average absolute residual correlation (range) of 0.248 (−0.279 to 0.476). Parameter estimates for the structural components in the model are presented in Figure 1.

Omitting the cross-loadings in the hierarchical model and using informative, small-variance priors for the residual covariances δ ~ *IW*(0, 56) in the measurement model also yields satisfactory model fit for both the female (PP*p* = 0.634, Δ observed and replicated χ^2^ 95% CI [−168.305, 123.630]) and male groups (PP*p* = 0.800, Δ observed and replicated χ^2^ 95% CI [−215.656, 88.242]), in contrast to a model that only has cross-loadings with even less strict prior variances [λ~ *N*(0, 0.09) corresponding to 95% cross-loading limits of ±0.59], which leads to a low PP*p* (<0.05). However, in the structural model for the female group, all target loadings are significant, although the loading of the psychomotor overexcitability factor on the latent variable of positive developmental potential must be considered small (λ = 0.261). Not permitting cross-loadings in the measurement model considerably increases the number of non-trivial residual covariances (158 for females, and 124 for males) and inflates parameter estimates.

In Bayesian analysis, the deviance information criterion (DIC) can be used for the purpose of comparing different models, where the model with the lowest DIC value is preferably selected (Spiegelhalter et al., 2002). The DIC values generated by the first higher order model and the second hierarchical model were 40,490.867 and 40,459.584 for the female group, and 25,991.245 and 25,956.312 for the male group, respectively. Although the difference in DIC is small, the models that only included residual covariances produced the smallest DIC values. However, the models with more constraints led to considerably more significant residual covariances (and, as a consequence, lower DIC values), making model comparison more difficult. Our results correspond to previous studies mentioning higher loadings on a second-order latent variable and inflated first-order factor correlations in the case of more strict models (Golay et al., 2013).

### Multiple-group BSEM-based alignment with approximate measurement invariance

Table 7 presents the results of the BSEM multiple-group approximate measurement invariance analysis with zero-means and decreasing variances for the prior distributions of differences in factor loadings and intercepts across gender. The extent to which the prior variance can be reduced is monitored through the PP*p*. “If the prior variance is small relative to the magnitude of non-invariance, PPP will be lower than if the prior variance corresponds better to the magnitude of non-invariance” (Muthén and Asparouhov, 2013a, p. 21). Analyses were executed for each overexcitability factor, since configural invariance had already been established (cf., BSEM models with informative, small-variance priors for cross-loadings and residual covariances). For the intellectual overexcitability data a prior variance for the measurement parameters of 0.01 results in a PP*p* of 0.540. Decreasing the prior variance does not alter the PP*p* substantially. A prior variance of 0.000000001—resulting in an excellent PP*p* of 0.559—entails a strong informative prior belief that 95% of the distribution of non-invariance is situated within the range of [−0.000062; +0.000062], which represents an extremely small range around zero.

**Table 7:** **Model fit coefficients of multiple-group BSEM-based alignment with approximate measurement invariance per overexcitability factor using varying prior variances**.

**Prior variance σ^2^**	**Intellectual OE**		**Imaginational OE**		**Emotional OE**		**Sensual OE**		**Psychomotor OE**	
	**PP *p***	**95% CI**	**PP *p***	**95% CI**	**PP *p***	**95% CI**	**PP *p***	**95% CI**	**PP *p***	**95% CI**
0.01	0.540	−42.660–52.130	0.392	−38.731–46.347	0.500	−49.966–38.545	0.598	−48.852–43.248	0.518	−59.937–40.278
0.001	0.589	−51.289–40.207	0.275	−32.969–56.720	0.441	−45.894–44.876	0.491	−38.249–43.758	0.235	−34.540–51.567
0.0001	0.578	−47.817–40.790	0.232	−35.467–72.492	0.402	−45.185–48.596	0.455	−34.207–48.716	0.157	−29.045–62.081
0.00001	0.559	−47.853–44.884	0.232	−37.887–72.639	0.392	−46.726–47.701	0.455	−33.556–50.392	0.157	−28.902–63.374
0.000001	0.559	−48.030–46.511	0.232	−39.102–70.930	0.402	−46.654–47.810	0.455	−33.668–51.832	0.167	−29.122–63.611
0.0000001	0.559	−48.083–47.011	0.225	−33.007–59.420	0.402	−46.508–47.884	0.455	−33.740–52.255	0.167	−29.234–63.665
0.00000001	0.559	−48.098–47.165	0.225	−33.042–59.346	0.402	−46.448–47.911	0.446	−33.761–52.384	0.167	−29.274–63.680
0.000000001	0.559	−48.103–47.214	0.225	−33.053–59.322	0.402	−46.428–47.920	0.446	−33.768–52.425	0.157	−29.287–63.685

*BSEM, Bayesian structural equation modeling; OE, overexcitability; PP p, posterior predictive probability; CI, confidence interval*.

Scalar invariance, as characterized by invariant factor loadings and measurement intercepts, is a prerequisite to compare factor means across groups (Vandenberg and Lance, 2000; Millsap, 2011; Muthén and Asparouhov, 2013c). For intellectual overexcitability, the factor loadings and intercepts are all invariant, regardless of the simulated prior variance, and none of the groups show a significantly (at the 5% significance level) different factor mean. For the construct of imaginational overexcitability, the use of a prior variance of 0.01 and 0.000000001 generates PP*p*s of 0.392 and 0.225, respectively. The factor loadings and intercepts are all invariant, and none of the groups show a significantly different factor mean. For emotional overexcitability, a prior variance of 0.01, 0.001, and smaller, results in PP*p*s of 0.500, 0.441, and 0.402, respectively. The factor loadings and intercepts are all invariant, and the male group shows a significantly smaller factor mean. For sensual overexcitability, a prior variance of 0.01, 0.001, and smaller, results in PP*p*s of 0.598, 0.491, and ~0.450, respectively. The factor loadings and intercepts are all invariant, and the male group shows a significantly smaller factor mean. For the psychomotor overexcitability data a prior variance of 0.01 results in a PP*p* of 0.518. The factor loadings are all invariant, although the intercept of item y50 (“I thrive on intense physical activity, e.g., fast games and sports”) is non-invariant across gender. Decreasing the prior variance to 0.001 or smaller, still produces an acceptable PP*p* of 0.235 and ~0.160, respectively, and leads the non-invariance of the intercept of y50 to disappear. The factor loadings and intercepts are all invariant and the female group shows a significantly smaller factor mean.

According to the acceptable PP*p*s and corresponding CIs even under strict conditions (i.e., the use of prior distributions with extremely small variances of 0.000000001), we may conclude that approximate scalar measurement invariance is supported by the data for each of the overexcitability latent variables.

## Discussion

The first aim of this study was to validate the factorial structure of the OEQ-II using Bayesian estimation in comparison with the frequentist approach to validation. To this end, the new concept of BSEM, as presented by Muthén and Asparouhov (2012), was applied with informative, small-variance priors for cross-loadings and residual covariances, which better reflects substantive theory. The analysis yielded positive results regarding the factorial validity of the OEQ-II, in contrast to the maximum likelihood CFA and EFA models which could not generate a satisfactory model fit. The hypothesized factor loading pattern was not fully recovered by the EFA results, due to the existence of many minor residual covariances. Freeing all residual covariances in a frequentist analysis would lead to a non-identified model. Alternatively, modifying the model using modification indices may capitalize on chance (MacCallum et al., 1992), with a large risk of model misspecification (Muthén and Asparouhov, 2013b). However, Bayesian analysis allows for all residual covariances to be inserted into the model using zero-mean, small-variance prior distributions, therefore overriding the problem of non-identification. Moreover, the BSEM approach “informs about model modification when all parameters are freed and does so in a single-step analysis” (Muthén and Asparouhov, 2012, p. 313). BSEM led to good model fit, as evaluated by means of Posterior Predictive Checking, which is less susceptible to slight, negligible model misspecifications compared to the chi-square statistic for assessing model fit (Muthén and Asparouhov, 2012). It also led to less inflated factor correlations compared to CFA, and satisfactory loading pattern recovery with substantial target loadings.

However, one major factor loading, namely the loading of item y2 (“I can take difficult concepts and translate them into something more understandable”) on the latent factor of intellectual overexcitability, was not found to be substantive (although the standardized coefficient of the loading was the largest for this item). Although the content of y2 is consistent with the content of the other items that load significantly on the latent variable of intellectual overexcitability, perhaps a higher standard is required to yield the response of agreement. The level of conceptual difficulty is not defined in more detail and can be interpreted differently by various people. The study by Van den Broeck et al. (2014) also revealed a low but significant factor loading (λ = 0.33) for the respective item. Future validation studies of the OEQ-II will have to affirm how y2 compares relative to the other factor indicators and in relation to the construct of intellectual overexcitability.

Regarding the results of the higher order model, we may conclude that the construct of psychomotor overexcitability, as captured by the OEQ-II, behaves differently to intellectual, imaginational, emotional, and sensual overexcitability. The latter forms of overexcitability all load substantially on the superordinate latent variable of positive developmental potential. According to Dabrowski's theory, the presence of only psychomotor and/or sensual overexcitability in an individual hinders advanced development (Dabrowski, 1972; Mendaglio, 2012). However, according to the results of this study, the construct of sensual overexcitability is strongly related to three of the most important drivers of personality growth. Piechowski (2013, p. 105) stated that under emotional tension, psychomotor overexcitability can be manifested as “compulsive talking and chattering, impulsive actions, nervous habits (tics, nail biting), workaholism, acting out,” and sensual overexcitability can be expressed as “overeating, self-pampering, sex as pacifier and escape, buying sprees, desire to be in the limelight.” Only one item of the OEQ-II is related to the expression of psychomotor or sensual overexcitability under difficult emotional circumstances (i.e., “When I am nervous, I need to do something physical”). All of the items representing sensual overexcitability are expressed in a positive way, and are indicative of a very perceptive personality, as are the other three forms of overexcitability which are considered essential to advanced personality development. The 40 items of the OEQ-II representing intellectual, imaginational, emotional, and sensual overexcitability seem to be indicative of a conscious, complex, creative, deeply emotionally engaged, sensitive, and perceptive personality with a strong susceptibility to wonder. Psychomotor overexcitability, as represented by the OEQ-II, does not have that same kind of spirit, but is more neutral and related to intense physical activity and competitiveness. Mendaglio and Tillier (2006) rightly emphasize the importance of further elaborating the empirical research on developmental potential by incorporating specific talents and abilities, dynamisms, and features of the environment alongside overexcitabilities in future studies. The results of this study also demonstrate the importance of more thoroughly examining the specific, possibly mediational role of psychomotor overexcitability in the process of personality growth, as viewed from the perspective of Dabrowski's theory.

Results of simulation studies indicate that approximate measurement invariance with highly precise priors outperforms full and partial measurement invariance in the case of (many) small differences in measurement parameters across groups (van de Schoot et al., 2013). In our study, which applied BSEM-based alignment with approximate measurement invariance, the absence of non-invariant factor loadings and intercepts across gender was indicative of the psychometric quality of the OEQ-II. The results of our study revealed a significantly higher score for females on emotional and sensual overexcitability, and a significantly lower score on the construct of psychomotor overexcitability compared to males. These results are mostly consistent with the findings of the previous studies mentioned above. However, no difference could be established in the level of intellectual overexcitability across both gender groups. The rather intellectual homogeneity of the sample may explain this result.

BSEM is an innovative and flexible approach to statistics, allowing the application of zero-mean, small-variance priors for cross-loadings, residual covariances, and differences in measurement parameters across groups, which leads to better model fit and less overestimation of factor correlations compared to CFA (which postulates exact parameter constraints and is usually too strict; Muthén, 2013; Muthén and Asparouhov, 2013a; Fong and Ho, 2014).

More generally, the Bayesian approach to statistics has many advantages over the frequentist approach. Bayesian analysis makes it possible to incorporate prior knowledge—with different degrees of uncertainty, as indicated by the variance of the prior distribution—into parameter estimation, and is well suited for testing complex, non-linear models with non-normal distributions, regardless of sample size (Kruschke et al., 2012). Even in the case of very limited prior knowledge (non-informative prior) with little influence on the posterior distribution, the Bayesian credibility interval nevertheless allows direct probability statements about the parameter values given the data (Kruschke et al., 2012).

With regard to the limitations of this study, we have to note that although the BSEM approach to factorial validation and measurement invariance analysis better represents substantive theory and avoids the need for a long series of model modifications with a substantial risk of misspecification, it is an innovative method that requires further research. Muthén and Asparouhov (2012) rightly emphasize the difficulty of balancing the need for small-variance priors for cross-loadings and small prior variances for residual covariances, which is supported by the results of the sensitivity analysis of the higher order model in this study. Moreover, the degree of susceptibility of the PP*p* to model misspecification warrants further research. This is of major importance given the strong influence of small-variance priors on the posterior parameter distributions, even in medium-sized samples. Furthermore, the susceptibility of the PP*p* to specific model features, the number of variables, and variable distributions needs to be investigated in more detail (Muthén and Asparouhov, 2012). One reviewer rightly stressed the limitation of the use of rather small sample sizes in this study—especially with regard to the male sample—according to standard criteria applied in conventional CFA and SEM analyses. Although the PP*p* has been found to perform better with small sample sizes than the maximum likelihood chi-square statistic, and to be less prone to negligible model misspecifications (Muthén and Asparouhov, 2012), the susceptibility of the PP*p* to the number of observations as well as the performance of BSEM estimation under varied sample sizes (and model features) should definitely be examined further. Future BSEM studies should investigate which sample size is required according to the number of degrees of freedom included in the model in order to ensure optimum performance. However, preliminary studies indicate that Bayesian SEM performs better with small sample sizes than does maximum likelihood SEM (Lee and Song, 2004).

In any case, using Bayesian analysis, either as a pragmatic or meta-analytic approach, it is crucial to perform sensitivity analyses which investigate the effect of varying the means and variances of prior distributions on the parameter estimates and model fit. The performance of the alignment optimization method under varied conditions also needs to be investigated further, as it represents a novel technique for measurement invariance analysis under certain assumptions.

A second limitation of this study is the use of a convenience sample to simultaneously investigate the factorial structure of the OEQ-II, as well as approximate measurement invariance of factor loadings and intercepts across gender. Future studies should preferably use independent randomized samples to cross-validate the OEQ-II and investigate (approximate) measurement invariance across varied conditions.

Apart from this, the results of our study coincide with the findings of the study by Van den Broeck et al. (2014), and are supportive of the psychometric quality of the OEQ-II.

The Mplus scripts for the main BSEM analyses in this study are available as Supplementary Material.

## Author contributions

ND: study design, data analysis, drafting the manuscript, approving the final version of the manuscript for submission, accountable for all aspects of the work. PV: data acquisition, critically revising the manuscript, approving the final version of the manuscript for submission, accountable for all aspects of the work.

### Conflict of interest statement

The authors declare that the research was conducted in the absence of any commercial or financial relationships that could be construed as a potential conflict of interest.

## Footnotes

^1^The Bayesian credibility interval can be retrieved directly from the percentiles of the posterior probability distribution of the model parameters. Using the posterior distribution percentiles, it is possible to determine directly the probability that a population parameter value is situated within a specific interval. In this study, which used Bayesian analysis as a pragmatic approach, a (null) hypothesis testing perspective (Arbuckle, 2013; Zyphur and Oswald, 2015) was used in parameter estimation by evaluating whether the 95% credibility interval of the model parameters encompassed zero. If the posterior probability interval of a particular parameter does not contain zero, the null (condition) can be rejected as implausible, and as a consequence, the parameter is considered significant (which is indicated by a one-tailed Bayesian *p*-value below 0.05). A hypothesis testing perspective was also used in assessing model fit (Levy, 2011).

^2^As previously mentioned in footnote 1, a hypothesis testing perspective was used in this study. In Bayesian parameter estimation, the term “significant” is used by the authors to indicate that the 95% Bayesian credibility interval of a particular parameter did not cover zero.

## References

[B1] Arbuckle J. L. (2013). IBM SPSS Amos 22 User's Guide. Chicago, IL: Amos Development Corporation.

[B2] Asparouhov T. Muthén B. (2009). Exploratory structural equation modeling. Struct. Equ. Modeling 16, 397–438. 10.1080/10705510903008204

[B3] Asparouhov T. Muthén B. (2010). Bayesian Analysis Using Mplus: Technical Implementation. Technical Appendix. Los Angeles, CA: Muthén & Muthén.

[B4] Asparouhov T. Muthén B. (2014). Multiple-group factor analysis alignment. Struct. Equ. Modeling 21, 495–508. 10.1080/10705511.2014.919210

[B5] Bentler P. M. (1990). Comparative fit indexes in structural models. Psychol. Bull. 107, 238–246. 10.1037/0033-2909.107.2.238 2320703

[B6] Bolstad W. M. (2007). Introduction to Bayesian Statistics, 2nd Edn. Hoboken, NJ: Wiley.

[B7] Botella M. Fürst G. Myszkowski N. Storme M. Pereira Da Costa M. Luminet O. (2015). French validation of the overexcitability questionnaire 2: psychometric properties and factorial structure. J. Pers. Assess. 97, 209–220. 10.1080/00223891.2014.938750 25090583

[B8] Bouchet N. Falk R. F. (2001). The relationship among giftedness, gender, and overexcitability. Gift. Child Q. 45, 260–267. 10.1177/001698620104500404

[B9] Browne M. W. Cudeck R. (1989). Single sample cross-validation indices for covariance structures. Multivariate Behav. Res. 24, 445–455. 10.1207/s15327906mbr2404_4 26753509

[B10] Carman C. A. (2011). Adding personality to gifted identification: relationships among traditional and personality-based constructs. J. Adv. Acad. 22, 412–446. 10.1177/1932202X1102200303

[B11] Cole D. A. Ciesla J. A. Steiger J. H. (2007). The insidious effects of failing to include design-driven correlated residuals in latent-variable covariance structure analysis. Psychol. Methods 12, 381–398. 10.1037/1082-989x.12.4.381 18179350

[B12] Dabrowski K. (1964). Positive Disintegration. Boston, MA: Little Brown.

[B13] Dabrowski K. (1972). Psychoneurosis is Not an Illness. London: Gryf Publications.

[B14] Daniels S. Piechowski M. M. (2009). Embracing intensity: overexcitability, sensitivity, and the developmental potential of the gifted, in Living With Intensity: Understanding the Sensitivity, Excitability, and Emotional Development of Gifted Children, Adolescents, and Adults, eds Daniels S. Piechowski M. M. (Scottsdale, AZ: Great Potential Press), 3–17.

[B15] Falk R. F. Lind S. Miller N. B. Piechowski M. M. Silverman L. K. (1999). The Overexcitability Questionnaire – Two (OEQ-II): Manual, Scoring System, and Questionnaire. Denver, CO: Institute for the Study of Advanced Development.

[B16] Falk R. F. Miller N. B. (2009). Building firm foundations: research and assessments, in Living with Intensity: Understanding the Sensitivity, Excitability, and Emotional Development of Gifted Children, Adolescents, and Adults, eds Daniels S. Piechowski M. M. (Scottsdale, AZ: Great Potential Press), 239–259.

[B17] Falk R. F. Yakmaci-Guzel B. Chang A. H. Pardo de Santayana Sanz R. Chavez-Eakle R. A. (2008). Measuring overexcitability: replication across five countries, in Dabrowski's Theory of Positive Disintegration, ed Mendaglio S. (Scottsdale, AZ: Great Potential Press), 183–199.

[B18] Fong T. C. Ho R. T. (2014). Testing gender invariance of the Hospital Anxiety and Depression Scale using the classical approach and Bayesian approach. Qual. Life Res. 23, 1421–1426. 10.1007/s11136-013-0594-3 24307211

[B19] Gelman A. Carlin J. B. Stern H. S. Dunson D. B. Vehtari A. Rubin D. B. (2014). Bayesian Data Analysis, 3rd Edn. New York, NY: Chapman & Hall.

[B20] Gelman A. Rubin D. B. (1992). Inference from iterative simulation using multiple sequences. Stat. Sci. 7, 457–472. 10.1214/ss/1177011136

[B21] Geman S. Geman D. (1984). Stochastic relaxation, Gibbs distributions and the Bayesian restoration of images. IEEE Trans. Pattern Anal. Mach. Intell. 6, 721–741. 10.1109/TPAMI.1984.4767596 22499653

[B22] Golay P. Reverte I. Rossier J. Favez N. Lecerf T. (2013). Further insights on the French WISC–IV factor structure through Bayesian structural equation modeling. Psychol. Assess. 25, 496–508. 10.1037/a0030676 23148651

[B23] Harrison G. E. Van Haneghan J. P. (2011). The gifted and the shadow of the night: Dabrowski's overexcitabilities and their correlation to insomnia, death anxiety, and fear of the unknown. J. Educ. Gift. 34, 669–697. 10.1177/016235321103400407

[B24] He W.-J. Wong W.-C. (2014). Greater male variability in overexcitabilities: domain-specific patterns. Pers. Individ. Dif. 66, 27–32. 10.1016/j.paid.2014.03.002

[B25] Hu L. Bentler P. M. (1995). Evaluating model fit, in Structural Equation Modeling: Concepts, Issues and Applications, ed Hoyle R. H. (Thousand Oaks, CA: Sage), 76–99.

[B26] Kaplan D. Depaoli S. (2012). Bayesian structural equation modeling, in Handbook of Structural Equation Modeling, ed Hoyle R. H. (New York, NY: The Guilford Press), 650–673.

[B27] Kruschke J. K. (2015). Doing Bayesian Data Analysis, Second Edition: A Tutorial with R, JAGS, and Stan. Waltham, MA: Academic Press; Elsevier.

[B28] Kruschke J. K. Aguinis H. Joo H. (2012). The time has come: Bayesian methods for data analysis in the organizational sciences. Organ. Res. Methods 15, 722–752. 10.1177/1094428112457829

[B29] Lee S.-Y. (2007). Structural Equation Modeling: A Bayesian Approach. West Sussex, UK: Wiley.

[B30] Lee S.-Y. Song X.-Y. (2004). Evaluation of the Bayesian and maximum likelihood approaches in analyzing structural equation models with small sample sizes. Multivariate Behav. Res. 39, 653–686. 10.1207/s15327906mbr3904_4 26745462

[B31] Levy R. (2011). Bayesian data-model fit assessment for structural equation modeling. Struct. Equ. Modeling 18, 663–685. 10.1080/10705511.2011.607723

[B32] MacCallum R. C. Roznowski M. Necowitz L. B. (1992). Model modifications in covariance structure analysis: the problem of capitalizing on chance. Psychol. Bull. 111, 490–504. 10.1037/0033-2909.111.3.490 16250105

[B33] MacKinnon D. P. (2008). Introduction to Statistical Mediation Analysis. New York, NY: Erlbaum.

[B34] Marsh H. W. Muthén B. Asparouhov T. Lüdtke O. Robitzsch A. Morin A. J. S. (2009). Exploratory structural equation modeling, integrating CFA and EFA: application to students' evaluations of university teaching. Struct. Equ. Modeling 16, 439–476. 10.1080/10705510903008220

[B35] Marsh H. W. Nagengast B. Morin A. J. (2013). Measurement invariance of big-five factors over the life span: ESEM tests of gender, age, plasticity, maturity, and la dolce vita effects. Dev. Psychol. 49, 1194–1218. 10.1037/a0026913. 22250996

[B36] Mendaglio S. (2008). Dabrowski's theory of positive disintegration: a personality theory for the 21st century, in Dabrowski's Theory of Positive Disintegration, ed Mendaglio S. (Scottsdale, AZ: Great Potential Press), 13–40.

[B37] Mendaglio S. (2012). Overexcitabilities and giftedness research: a call for a paradigm shift. J. Educ. Gift. 35, 207–219. 10.1177/0162353212451704

[B38] Mendaglio S. Tillier W. (2006). Dabrowski's Theory of positive disintegration and giftedness: overexcitability research findings. J. Educ. Gift. 30, 68–87. 10.1177/016235320603000104

[B39] Miller N. B. Falk R. F. Huang Y. (2009). Gender identity and the overexcitability profiles of gifted college students. Roeper Rev. 31, 161–169. 10.1080/02783190902993920

[B40] Millsap R. E. (2011). Statistical Approaches to Measurement Invariance. New York, NY: Routledge.

[B41] Muthén B. (2013). Advances in latent variable modeling using Mplus version 7, in Workshop at the Modern Modeling Methods Conference (Washington, DC: University of Connecticut, and at the APS Convention). Available online at: www.statmodel.com

[B42] Muthén B. Asparouhov T. (2012). Bayesian structural equation modeling: a more flexible representation of substantive theory. Psychol. Methods 17, 313–335. 10.1037/a0026802 22962886

[B43] Muthén B. Asparouhov T. (2013a). BSEM Measurement Invariance Analysis. Mplus Web Notes: No.17. Available online at: www.statmodel.com

[B44] Muthén B. Asparouhov T. (2013b). Late-Breaking News: Some Exciting New Methods. Keynote Address at the Modern Modeling Methods Conference, University of Connecticut. Available online at: www.statmodel.com

[B45] Muthén B. Asparouhov T. (2013c). New Methods for the Study of Measurement Invariance with Many Groups. Available online at: www.statmodel.com

[B46] Muthén L. K. Muthén B. O. (1998-2012). Mplus User's Guide, 7th Edn. Los Angeles, CA: Muthén & Muthén.

[B47] Muthén L. K. Muthén B. O. (2013). Version 7.1 Mplus Language Addendum. Available online at: www.statmodel.com

[B48] Piechowski M. M. (2013). A bird who can soar: overexcitabilities in the gifted, in Off the Charts: Asynchrony and the Gifted Child, eds Neville C. S. Piechowski M. M. Tolan S. S. (Unionville, NY: Royal Fireworks Press), 99–122.

[B49] Rinn A. N. Mendaglio S. Rudasill K. M. McQueen K. S. (2010). Examining the relationship between the overexcitabilities and self-concepts of gifted adolescents via multivariate cluster analysis. Gift. Child Q. 54, 3–17. 10.1177/0016986209352682

[B50] Scheines R. Hoijtink H. Boomsma A. (1999). Bayesian estimation and testing of structural equation models. Psychometrika 64, 37–52. 10.1007/BF02294318

[B51] Silverman L. K. (2008). The theory of positive disintegration in the field of gifted education, in Dabrowski's Theory of Positive Disintegration, ed Mendaglio S. (Scottsdale, AZ: Great Potential Press), 157–173.

[B52] Siu A. F. Y. (2010). Comparing overexcitabilities of gifted and non-gifted school children in Hong Kong: does culture make a difference? Asia Pac. J. Educ. 30, 71–83. 10.1080/02188790903503601

[B53] Spiegelhalter D. J. Best N. G. Carlin B. P. van der Linde A. (2002). Bayesian measures of model complexity and fit. J. R. Stat. Soc. B 64, 583–639. 10.1111/1467-9868.00353

[B54] Steiger J. H. (1990). Structural model evaluation and modification: an interval estimation approach. Multivariate Behav. Res. 25, 173–180. 10.1207/s15327906mbr2502_4 26794479

[B55] Tieso C. L. (2007a). Overexcitabilities: a new way to think about talent? Roeper Rev. 29, 232–239. 10.1080/02783190709554417

[B56] Tieso C. L. (2007b). Patterns of overexcitabilities in identified gifted students and their parents: a hierarchical model. Gift. Child Q. 51, 11–22. 10.1177/0016986206296657

[B57] Treat A. R. (2006). Overexcitability in gifted sexually diverse populations. J. Adv. Acad. 17, 244–257. 10.4219/jsge-2006-413

[B58] Vandenberg R. J. Lance C. E. (2000). A review and synthesis of the measurement invariance literature: suggestions, practices, and recommendations for organizational research. Organ. Res. Methods 3, 4–70. 10.1177/109442810031002

[B59] Van den Broeck W. Hofmans J. Cooremans S. Staels E. (2014). Factorial validity and measurement invariance across intelligence levels and gender of the Overexcitabilities Questionnaire – II (OEQ-II). Psychol. Assess. 26, 55–68. 10.1037/a0034475 24079958

[B60] van de Schoot R. Kluytmans A. Tummers L. Lugtig P. Hox J. Muthén B. (2013). Facing off with Scylla and Charybdis: a comparison of scalar, partial, and the novel possibility of approximate measurement invariance. Front. Psychol. 4:770. 10.3389/fpsyg.2013.00770 24167495 PMC3806288

[B61] Warne R. T. (2011). An investigation of measurement invariance across genders on the Overexcitability Questionnaire–Two. J. Adv. Acad. 22, 578–593. 10.1177/1932202X11414821

[B62] Wirthwein L. Becker C. V. Loehr E. Rost D. H. (2011). Overexcitabilities in gifted and non-gifted adults: does sex matter? High Abil. Stud. 22, 145–153. 10.1080/13598139.2011.622944

[B63] Wirthwein L. Rost D. H. (2011). Focussing on overexcitabilities: studies with intellectually gifted and academically talented adults. Pers. Individ. Dif. 51, 337–342. 10.1016/j.paid.2011.03.041

[B64] Zyphur M. J. Oswald F. L. (2015). Bayesian estimation and inference: a user's guide. J. Manage. 41, 390–420. 10.1177/0149206313501200

