# Supplementary material for: Psychometric Evaluation of the Overexcitability Questionnaire-Two Applying Bayesian Structural Equation Modeling (BSEM) and Multiple-Group BSEM-Based Alignment with Approximate Measurement Invariance
Source: Front Psychol. 2015 Dec 24;6:1963. doi: 10.3389/fpsyg.2015.01963 (PMC4689874; doi:10.3389/fpsyg.2015.01963)
Supplement: Supplementary file 1 [file Mplus_Scripts.PDF]

Supplementary Material

Psychometric evaluation of the Overexcitability Questionnaire-Two applying Bayesian Structural Equation Modeling (BSEM) and multiple-group BSEM-based alignment with approximate measurement invariance

Niki De Bondt* and Peter Van Petegem

*** Correspondence:**

Niki De Bondt, *e-mail:* niki.debondt@uantwerpen.be

1 BSEM with informative, small-variance priors for cross-loadings and residual covariances – Mplus code

```
TITLE:      BSEM on OEQ-II data – females
DATA:       FILE =  Data.dat;
VARIABLE:   NAMES =
            Gender
            y1-y50;
            usevariables y1-y50;
            ! 5-point scale from 1 "Not at all like me" to 5 "Very much like me"
            missing are all (-99);
            ! grouping is Gender (0=g1 1=g2);
            ! Gender = 0 for males and 1 for females
            useobs = Gender eq 1;
DEFINE:     standardize y1-y50;
ANALYSIS:   estimator = Bayes;
            process = 2;
            chain = 1;
            fbiter = 100000;
            thin=10;
MODEL:      fi by y1-y10*;
            fim by y11-y20*;
            fe by y21-y30*;
            fs by y31-y40*;
            fps by y41-y50*;
            fi-fps with fi-fps;
            fi-fps @1;
            ! x-loadings:
            fi by y11-y50*0 (i1-i40);
            fim by y1-y10*0 (im1-im10)
            y21-y50*0 (im11-im40);
            fe by y1-y20*0 (e1-e20)
```

```
                y31-y50*0 (e21-e40);
                fs by y1-y30*0 (s1-s30)
                y41-y50*0 (s31-s40);
                fps by y1-y40*0 (ps1-ps40);
                ! residual covariances:
                y1-y50 (p1-p50);
                y1-y50 with y1-y50 (p51-p1275);
MODEL PRIORS:
                i1-i40~N(0,0.01);
                im1-im40~N(0,0.01);
                e1-e40~N(0,0.01);
                s1-s40~N(0,0.01);
                ps1-ps40~N(0,0.01);
                p1-p50~IW(1,56);
                p51-p1275~IW(0,56);
OUTPUT:         tech1 tech8 stdy;
PLOT:           type = plot2;
```

2 Multiple-group BSEM-based alignment with approximate measurement invariance – Mplus code

```
TITLE:      BSEM MI on OEQ-II data – intellectual overexcitability
DATA:       FILE = Data MI.dat;
VARIABLE:   NAMES = u y1-y50 group;
            USEVARIABLES = y1-y10 group;
            missing are all (-99);
            CLASSES = c(2);
            KNOWNCLASS = c(group = 1-2);
ANALYSIS:   TYPE =MIXTURE;
            ESTIMATOR = BAYES;
            process = 2;
            biter = (1000);
            thin = 10;
            align = fixed(BSEM);
MODEL:      %OVERALL%
            f BY y1-y10* (lam#_1-lam#_10);
            [y1-y10] (nu#_1-nu#_10);
            y1-y10 (p#_1-p#_10);
            y1-y10 with y1-y10;
MODEL PRIORS:
            do(1,10) diff(lam1_#-lam2_#)~N(0,0.01);
            do(1,10) diff(nu1_#-nu2_#)~N(0,0.01);
            do(1,2) p#_1~iw(1,16);
            do(1,2) p#_2~iw(1,16);
            do(1,2) p#_3~iw(1,16);
            do(1,2) p#_4~iw(1,16);
            do(1,2) p#_5~iw(1,16);
            do(1,2) p#_6~iw(1,16);
            do(1,2) p#_7~iw(1,16);
            do(1,2) p#_8~iw(1,16);
            do(1,2) p#_9~iw(1,16);
            do(1,2) p#_10~iw(1,16);
OUTPUT:     TECH1 TECH8;
PLOT:       TYPE =PLOT2;
```
